# Clinical Applications of Electrical Conductivity Imaging Using MRI

**DOI:** 10.1002/jmri.70279

**Published:** 2026-03-07

**Authors:** Stefano Mandija, Khin Khin Tha, Nitish Katoch, Cihan Göksu, Ulrich Katscher, Rosalind Sadleir, Kyu‐Jin Jung, Jierong Luo, Ilias I. Giannakopoulos, Dong‐Hyun Kim, Karin Shmueli, Riccardo Lattanzi, Yusuf Ziya Ider, Axel Thielscher, Cornelis van den Berg

**Affiliations:** ^1^ Computational Imaging Group for MR Therapy and Diagnostic, Department of Radiotherapy, Center for Image Sciences University Medical Center Utrecht Utrecht the Netherlands; ^2^ Global Center for Biomedical Science and Engineering, Hokkaido University Faculty of Medicine Sapporo Japan; ^3^ Philips Healthcare Seoul Republic of Korea; ^4^ Department of Electrical and Electronics Engineering İzmir Institute of Technology Izmir Turkey; ^5^ Nekodu Teknoloji Ltd İzmir Türkiye; ^6^ Philips Innovative Technologies Hamburg Germany; ^7^ School of Biological and Health Systems Engineering Arizona State University Tempe Arizona USA; ^8^ Department of Electrical and Electronic Engineering Yonsei University Seoul Republic of Korea; ^9^ Department of Electrical Engineering and Computer Sciences University of California, Berkeley Berkeley California USA; ^10^ Department of Medical Physics and Biomedical Engineering University College London London UK; ^11^ Bernard and Irene Schwartz Center for Biomedical Imaging, Department of Radiology New York University Grossman School of Medicine New York New York USA; ^12^ Center for Advanced Imaging Innovation and Research (CAI^2^R), Department of Radiology New York University Grossman School of Medicine New York New York USA; ^13^ Department of Biomedical Engineering Baskent University Ankara Turkey; ^14^ Department of Health Technology Technical University of Denmark Kgs. Lyngby Denmark; ^15^ Danish Research Centre for Magnetic Resonance, Department of Radiology and Nuclear Medicine Copenhagen University Hospital Amager and Hvidovre Hvidovre Denmark

**Keywords:** conductivity MRI, current density imaging, electrical impedance tomography, electrical properties tomography, MR‐EPT

## Abstract

Magnetic resonance imaging (MRI) has emerged as a noninvasive technique for probing the electrical properties of biological tissues: electrical conductivity and relative permittivity. This review focuses on the electrical conductivity and provides a comprehensive overview of applications across both low‐ and high‐frequency regimes. At low frequencies (below 1 MHz), conductivity mapping primarily reflects tissue microstructure and ionic composition. In contrast, at high frequencies (around 100 MHz), tissue conductivity primarily reflects ionic composition. First, we summarize the theoretical foundations, technical developments, and reconstruction algorithms that underpin conductivity imaging, highlighting advances in magnetic resonance electrical impedance tomography, current density imaging, and electrical properties tomography. The main part of the article discusses preclinical and clinical applications, demonstrating the potential and possible roles of conductivity imaging in clinical settings, along with current challenges and emerging applications. Finally, we outline future directions toward integrating conductivity imaging into routine MRI protocols, with the potential to enhance diagnostic precision and therapeutic monitoring.

**Evidence Level:**

1.

**Technical Efficacy:**

Stage 3.

## 1 Introduction

Over the past few decades, noninvasive mapping of tissue electrical properties (conductivity [*σ*] and relative permittivity [*ε*_r_]) has become possible with magnetic resonance imaging (MRI). These properties describe how biological tissues respond to electric fields and are strongly influenced by the frequency of such applied electric fields.

Conductivity increases with frequency. At low frequencies (below 1 MHz), current primarily flows through extracellular fluids. As frequency rises, cell membranes become less resistive, allowing current to penetrate cells, which leads to higher conductivity. In contrast, relative permittivity decreases with increasing frequency. Biological tissues exhibit high permittivity at low frequencies due to polarization effects; as frequency increases, these effects diminish, resulting in lower permittivity values.

Besides frequency, electrical properties are also affected by other factors such as temperature, tissue composition, and physiological state. Variations in ion concentration and cellular architecture produce distinct frequency‐dependent profiles across different tissue types [1]. These unique signatures make electrical properties valuable biomarkers for noninvasive tissue characterization, disease detection, and monitoring response to therapies.

MRI research has primarily focused on conductivity imaging at low frequencies (below kilohertz, kHz) and high frequencies (around 100 MHz). This review provides an overview of conductivity mapping across both frequency ranges, with emphasis on clinical applications. A summary of the reviewed applications is provided in Table 1.

**TABLE 1:** Summary of pre‐clinical (PC) and clinical (C) applications of MRI‐based conductivity imaging across frequency ranges.

Frequency	Body region	Application	Type	Number of animals/subjects [literature reference]
Low (Hz‐kHz)	Brain	Current Density Imaging	C	13 [2]; 2 [3]; 8 [4]; 9 [5]; 5 [6]
		Conductivity Imaging	PC	2 beagles [7]
			C	2 [8]; 1 [9]; 1 [10]
		Stimulation	C	14 [11]
		Neuronal Current	C	4 [12]
		Abscess	PC	10 beagles [13]
		Degeneration	C	66 [14]
		Electroporation	PC	15 A/J mice [15]
	Legs	Conductivity Imaging	C	4 [16]
	Liver	RF ablation	PC	7 bovines [17]
High (64–400 MHz)	Brain	Healthy	C	1 [18]; 3 [19]; 2 [20]; 1 [21]; 18 [22]; 1 [23]; 1 [24]; 10 [25]
		Tumors	PC	6 F98 rats [26]; 10 ICR mice [27]; 9/14 BALB/c nude mice [28, 29]
			C	3 [20]; 14 [22]; 1 [23]; 1 [24]; 30 [30]; 6 [25]; 60 [31]; 2 [32]; 19 [33]
		Neuronal loss	PC	40 Sprague–Dawley rats [34]
		Ischemic injury/stroke	PC	14 Minipigs [35]
			C	1 [36]; 2 [37]; 27 [38]; 6 [39]
		Degeneration	C	74 [40]
		Demyelination	C	9 [25]; 1 [41]
		Infection/inflammation	C	1 [41]; 13 [42]
		Function	C	5 [43]; 5 [44]; 5 [45]; 30 [46]; 2 [47]; 11 [48]; 4 [49]
	Breast	Healthy	C	40 [50]
		Tumor	PC	6 Fisher rats [51]
			C	110 [52]; 40 [50]; 65 [53]; 81 [54]; 116 [55]; 44 [56]; 79 [57]
	Lungs	Lesions	C	5 [58]; 21 [59]; 20 [60]
	Heart	Function	PC	2 [61]
			C	6 [62]; 4 [63]
	Blood	Healthy	C	6 [62]
		Lymphoma	C	5 [33]
	Liver	Healthy	C	6 [64]
		Fibrosis	PC	45 Sprague–Dawley rats [65]
			C	24 [64]
	Cervix	Tumors	C	20 [66]; 5 [67]
	Prostate	Tumors	PC	Copenhagen rats: 7 [51]; 2 [68]
	Muscle	Function	C	10 [69]
	Spine	Demyelination	PC	1 Ezo Sika Deer [70]
		Disc degeneration	C	17 [71]; 54 [72]; 23 [73]

## 2 Low Frequencies Conductivity Imaging

### 2.1 Theory

**FIGURE 1:**
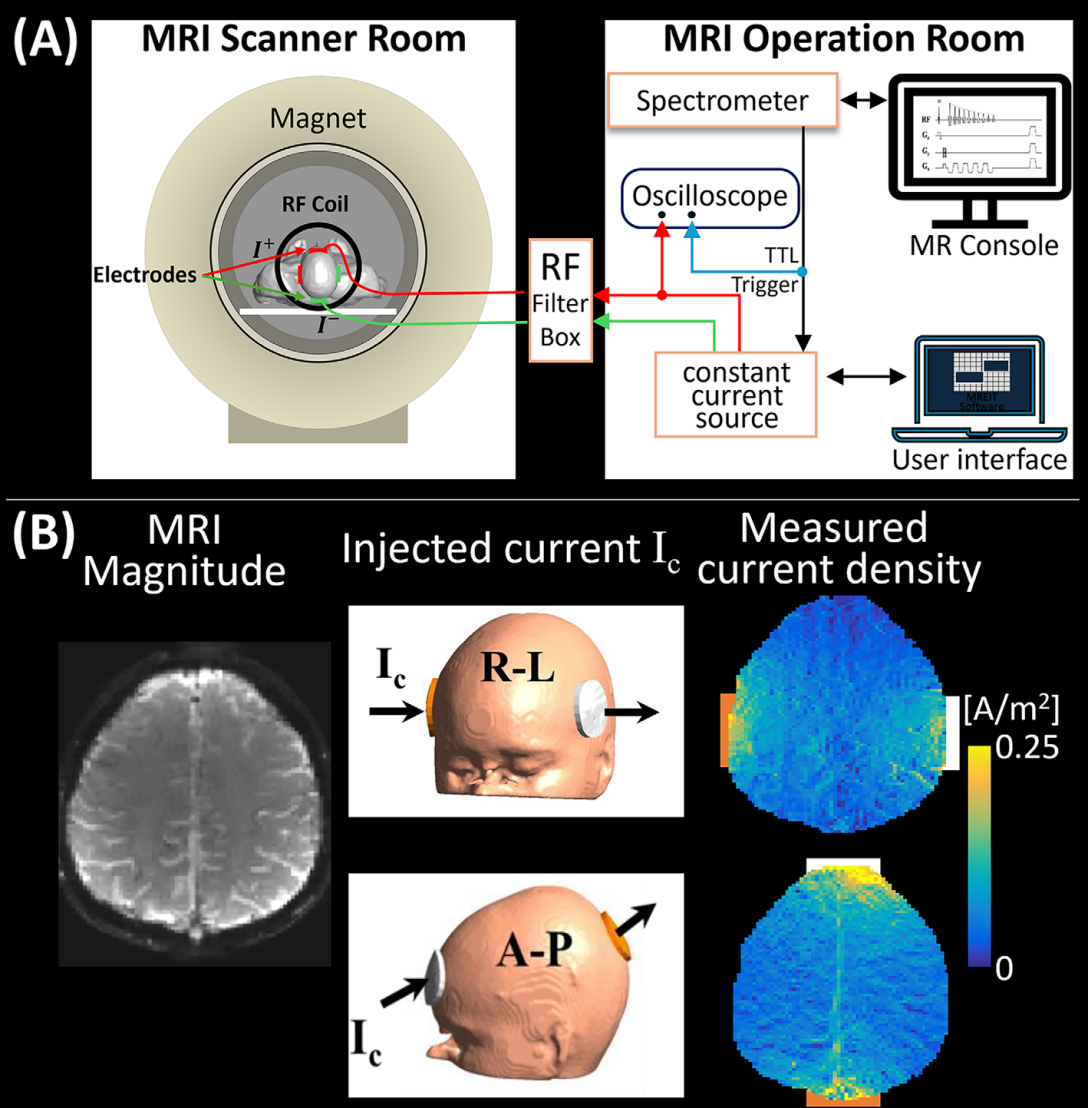
(A) Experimental setup used for MR‐EIT/CDI. Low‐amplitude currents are injected through surface electrodes to induce measurable magnetic fields used for conductivity or current density reconstruction. (B) Current density maps measured in the human head for right–left (R–L) and anterior–posterior (A–P) current injection montages, demonstrating in vivo estimation of intracranial current pathways and anisotropic current flow patterns (adapted from [2]).

Magnetic resonance current density imaging (MR‐CDI) and magnetic resonance electrical impedance tomography (MR‐EIT) utilize magnetic fields induced by externally applied electric currents (e.g., transcranial electric stimulation [TES]) to modulate the phase of MRI measurements. This phase modulation is then used to reconstruct current density and conductivity distributions (Figure 1) [74].

Low frequency electrical properties (typically < 100 Hz) can be measured using MRI by exploiting Ampere's law, which relates the current density distribution within an imaged object to the magnetic flux generated by the current flow. Since externally applied currents preferentially flow through the extracellular space, the resulting images of current density and conductivity are strongly influenced by cellular architecture and ionic composition.

Most in vivo applications have focused on the human brain [2, 3, 74]. In the simplest implementation, two MR acquisitions with opposite polarity currents are used to calculate the TES‐induced magnetic fields (∆*B*_ind_) by dividing the phase difference between the two MRI acquisitions by the phase sensitivity map. Due to safety constraints, in vivo current strengths are typically limited to ~1–2 mA. Consequently, research has focused on improving the signal‐to‐noise ratio (SNR) of MR methods by enhancing phase sensitivity without compromising image quality [4].

Yet, MRI provides only the component of the current‐induced magnetic flux density that is parallel to the MRI scanner's static magnetic field *B*_0_. Direct calculation of current density using Ampère's law requires knowledge of all three components of the induced field, necessitating repeated measurements with subject rotation inside the scanner, which is impractical for human imaging. Therefore, reconstruction algorithms that rely on a single field component are preferred [75]. From these ∆*B*_ind_ measurements, low frequency conductivity is then reconstructed. Alternative methods that do not rely on current injection into the body have also been presented (see Section 2.3).

### 2.2 Acquisitions

In MR‐CDI or MR‐EIT, currents are injected into subjects via surface electrodes, typically using rectangular waveforms whose polarity alternates in synchrony with the MR acquisition [2, 74]. At least two current injection profiles (preferentially orthogonally oriented) are needed to reconstruct projected current densities and conductivities, which benefit from volumetric ∆*B*_ind_ data [5].

The SNR of ∆*B*_ind_ scales with the SNR of the MR images, thus depending on the voxel size, the square root of the total signal acquisition time, the scanner field strength, and hardware [76]. In practice, the SNR of ∆*B*_ind_ is constrained by the maximum current strength applied. Additional limitations arise from susceptibility‐related signal dropouts, particularly near electrodes or paranasal sinuses [6], physiological noise, artifacts originating from blood and cerebrospinal fluid (CSF) flow [4], and respiratory and cardiac motion [2]. As a result, findings from phantom studies may not always translate directly to in vivo applications, making stringent quality assurance essential for human imaging. Beyond assessing baseline SNR levels without current injection, experiments involving current flow through wire loops around the body have proven valuable for identifying quality issues in ∆*B*_ind_ data, such as insufficient spoiling. In these cases, ∆*B*_ind_ can be accurately calculated from the wire loop geometry using the Biot–Savart law, offering a reliable ground‐truth even in vivo [4].

Numerous approaches were developed to maximize ∆*B*_ind_ sensitivity using spin‐echo (SE), gradient‐echo (GRE), echo planar imaging (EPI), hybrid, and steady‐state imaging techniques. GRE and SE methods often employ weighted multi‐echo acquisitions, as the associated SNR compromise is minimal compared to the preserved image quality enabled by higher bandwidths. Additional acquisition weighting in the phase‐encoding direction further enhances SNR efficiency and spatial resolution [2, 4].

Steady‐state approaches offer excellent phase sensitivity, but their complex signal evolution can limit the accuracy of ∆*B*_ind_ measurements. In practice, fully spoiled sequences tend to perform better [4]. Simultaneous multi‐slice acquisitions are well‐suited for improving volume coverage, whereas standard 3D acquisitions often suffer from amplified physiological noise, resulting in inferior performance [5]. Due to their intrinsic susceptibility to field drifts, these methods benefit from field monitoring techniques such as navigator echoes. Other strategies involve acquiring multiple averages of the same k‐space line with alternating readout polarity to mitigate drift effects. Alternatively, optimized EPI suffers less from low frequency noise in ∆*B*_ind_ images and achieves similar sensitivity in top brain slices, but is inferior in lower brain slices in feet‐head direction [6]. Multishot EPI has demonstrated excellent data quality in phantom studies while maintaining SNR [77].

As a result, state‐of‐the‐art MR‐EIT methods enable volumetric measurements with sub‐nanotesla sensitivity to current‐induced fields within ~10 min scan time [5]. In addition to ∆*B*_ind_ acquisitions, structural MRI is essential for constructing head models used in field simulations and for delineating cable paths to correct for stray magnetic fields [74].

### 2.3 Reconstruction

#### 2.3.1 Current Density

The most common current density reconstruction method, known as projected current density, uses first‐order spatial gradients of ∆*B*_ind_ measurements along with simulations performed using a model with uniform conductivity to estimate the current components orthogonal to *B*_0_ [78]. However, this approach captures only in‐plane currents, which is a significant limitation since currents flow throughout the brain, for example, for head TES [8]. Additionally, stray magnetic fields induced by current flow in the cables connected to the electrodes have been shown to clearly affect the measured data in humans, necessitating correction prior to further data processing [2, 74].

#### 2.3.2 Conductivity

Low frequency conductivity reconstruction methods were initially based on the calculation of the Laplacian of the measured ∆*B*_ind_ data [74]. However, this calculation severely amplifies measurement noise, limiting its use for human applications. To overcome this limitation, conductivity reconstructions based on the projected current flow were proposed, showing good correspondence between estimated and ground‐truth conductivity data in phantoms [78].

Alternatively, anisotropic conductivity can be reconstructed from the projected current density by exploiting diffusion tensor (DT) information from diffusion weighted imaging (DWI) [8]. Following the combination of conductivity and DT information to retrieve anisotropic conductivity, an electrodeless alternative to DT‐MR‐EIT, known as conductivity tensor imaging (CTI), retrieves low frequency anisotropic conductivity by combining high frequency conductivity measurements using either MR‐based electrical properties tomography (EPT) or water‐based EPT with DT measurements [7]. A recent study compared two anisotropic conductivity reconstruction approaches (DT‐MR‐EIT and electrodeless CTI) that both leverage the eigenvector relationship between diffusion and conductivity tensors [9]. The reconstructed conductivities fell within similar ranges and literature values, indicating good reconstruction accuracy [9]. On the other hand, other studies suggest that the limitations of the projected current density algorithm for human brain ∆*B*_ind_ data also make the conductivities estimated by DT‐MR‐EIT inaccurate and overly smoothed [3, 8].

Extensions of the original DT‐MR‐EIT approach—which traditionally requires two independent current administrations—have explored the feasibility of single‐current administration. Further developments have incorporated neural network‐based and deep learning (DL) methods to enable both single and multiple current reconstructions [10].

An alternative to DT‐MR‐EIT focuses on optimizing the conductivities of tissue compartments within head models to improve the fit between simulated and measured data [6, 8]. These simulations have shown significantly better agreement with measurements compared to those based on literature‐derived conductivity values. Notably, this method also estimates the conductivities of the skull and scalp—regions that do not yield reliable ∆*B*_ind_ MR data and are not captured by voxel‐wise reconstructions—but which critically influence TES current flow and electro/magneto‐encephalogram (EEG/MEG) source analysis outcomes [79].

One persistent challenge in current reconstruction approaches is the limited availability of data on the stability and typical range of estimated ohmic conductivities. Notably, low frequency conductivity values predicted by empirical models, for example models relying on water diffusion, are consistently lower than those recovered through multiple MR‐based measurements. This discrepancy underscores a broader issue: the absence of ground‐truth conductivity data for human in vivo brain imaging. As a result, there is currently no consensus on the accuracy of existing reconstruction methods for human brain low frequency conductivity imaging.

For a detailed overview of modeling approaches, current density, and conductivity reconstruction methods, we refer the reader to [74].

### 2.4 Clinical Applications

#### 2.4.1 Toward Realistic Head Models

A major impetus for developing accurate conductivity and current density imaging techniques is their potential application in constructing subject‐specific conductive brain models. Accurate volume conductor models of the head are essential for a wide range of clinical and research applications. These models significantly influence the accuracy of source localization in EEG and MEG, which is critical for detecting epileptogenic zones, identifying eloquent brain regions during presurgical planning, and diagnosing neurological disorders such as attention‐deficit/hyperactivity disorder (ADHD). Moreover, they play a key role in the precision of field simulations used to characterize and optimize the spatial targeting of brain stimulation techniques. These techniques aim to modulate neuronal excitability in specific brain regions for treating neuropsychiatric disorders [80], enhancing cognitive and working memory functions [81], and improving recovery following stroke [82].

Currently, volume conductor models typically rely on average conductivity values reported in literature. A limited number of validation studies have performed in vivo measurements in selected human patient populations and nonhuman primates using intracranial recordings [11]. While these studies generally show reasonable agreement between simulations and measurements, they also reveal notable mismatches in some individuals. Given that invasive recordings are not scalable, MR‐CDI and MR‐EIT offer promising noninvasive alternatives that could enable more frequent and individualized assessments of tissue conductivities.

#### 2.4.2 Neuronal Current Imaging

Blood‐oxygenation‐level‐dependent (BOLD) MRI contrast arises from an indirect effect: the differential magnetic susceptibility between oxygenated and deoxygenated hemoglobin near active brain regions. In addition to BOLD, MR magnitude and phase data are also sensitive to neural sources—a concept explored in neural current MRI (ncMRI) [83, 84]. However, predicted ncMRI signal or phase shifts are extremely small (~10^−12^ T), and reproducibility remains an issue because neuronal current‐induced signal attenuation was found to be less than 0.07% [12].

**FIGURE 2:**
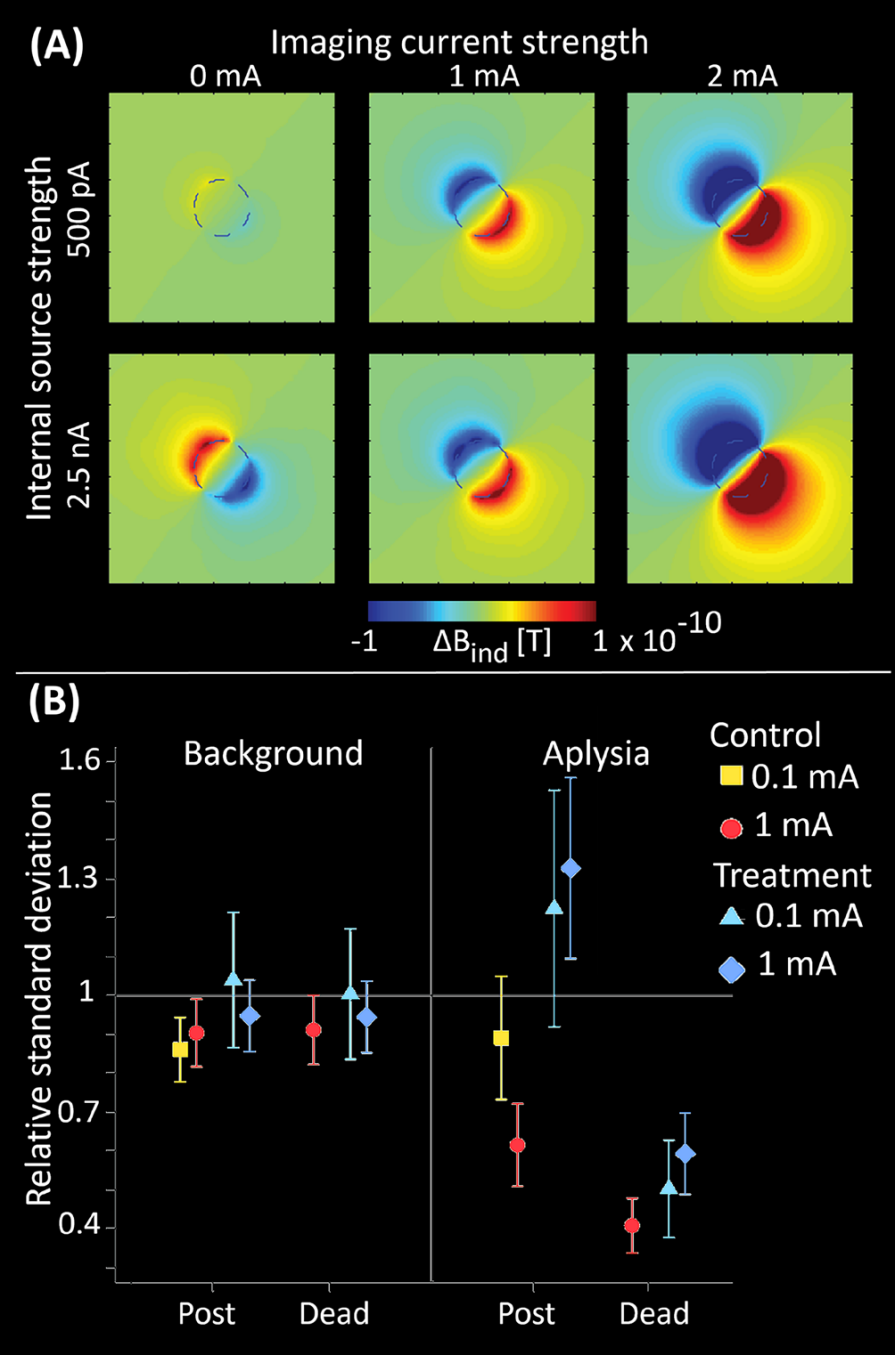
Estimated functional MR‐EIT (fMR‐EIT) signal changes and confirmation of fMR‐EIT in vitro. (A) Changes in Δ*B*_ind_ distributions over background field for a slice centered on the electrode plane for six different combinations of source and imaging currents using a voxel dimension of 70 × 70 × 500 micrometers cube (adapted from [83]). (B) Plot showing relative standard deviations, with 95% confidence intervals estimated via linear regression, in background or within Aplysia abdominal ganglion regions using 0.1 or 1 mA fMR‐EIT imaging of the ratio of standard deviations after neural activity was increased chemically (Post) or long after (Dead) to those measured before (Pre) treatment or control administrations. Standard deviations are shown for Post/Pre and Dead/Pre conditions for background MR‐EIT data, or Aplysia MR‐EIT data (adapted from [84]).

Yet, phase images can be influenced by apparent conductivity changes in active cells illuminated by externally applied low frequency currents. These changes are attributed to membrane conductance variations during action potentials. This phenomenon may be particularly useful for studying neuroplasticity in deep brain stimulation, where the stimulating current could also serve as a functional probe. Functional MR‐EIT studies and simulations have confirmed the existence of this effect in vitro at 11.75 T (Figure 2A) [83].

One in vitro study demonstrated that phase variability within excised abdominal ganglia of the sea slug *Aplysia californica* increased significantly in samples treated with a neuroirritant (saline solution with elevated potassium levels), compared to those treated with a standard solution or measured in background solution. Notably, these changes were observed in ganglia imaged with a 1 mA applied current but were absent at 100 μA, indicating a current‐dependent imaging effect (Figure 2B) [84].

#### 2.4.3 Emerging Applications

Although still in their infancy, low frequency conductivity imaging is finding promising clinical applications across a range of domains.

The first in vivo MR‐EIT feasibility study focused on imaging the human leg using a 3 T MRI scanner and injecting a 9 mA current in a form of 15 ms pulse without producing any painful sensation. The reconstructed low frequency conductivity images showed clear differences between subcutaneous adipose tissue, muscle, crural fascia, intermuscular septum, and bone inside the leg, demonstrating the in vivo feasibility of MR‐EIT [16].

**FIGURE 3:**
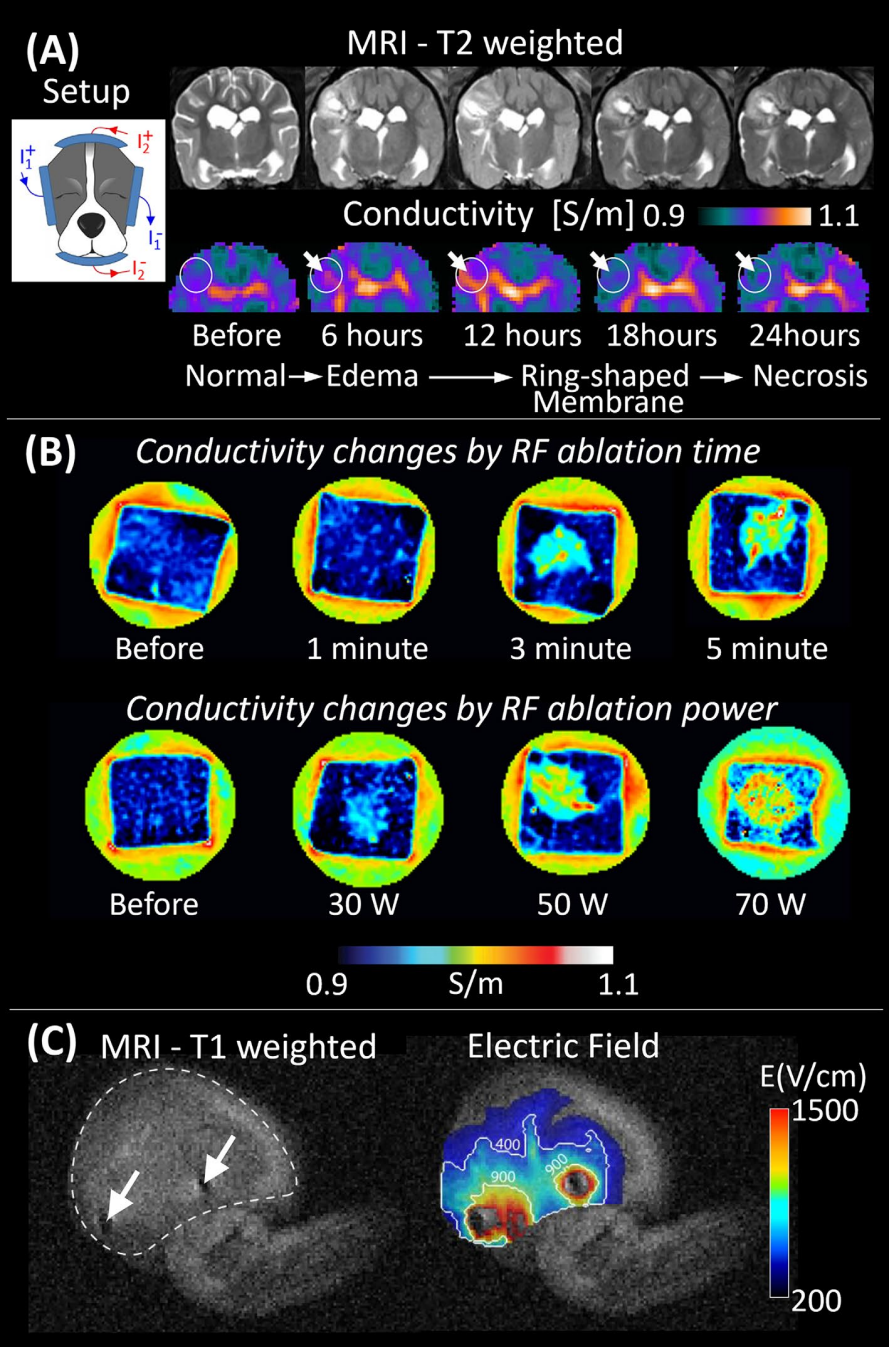
Example applications of low frequency conductivity imaging: (A) In vivo canine brain abscess model, showing T2 weighted and conductivity maps from baseline to 24 h post‐inoculation. Circles and arrows indicate temporal changes from early edema to central necrosis (adapted from [13]). (B) Tissue conductivity alterations during radiofrequency ablation as a function of ablation duration (top) and applied power (bottom), demonstrating localized conductivity increase with increasing thermal exposure (adapted from [17] by permission of Informa UK Limited, trading Taylor & Francis Group, https://www.tandfonline.com, 2013 Informa UK Ltd.). (C) In situ electric field distribution in a mouse tumor (dashed white line in T1 weighted image) obtained by means of MR‐EIT during electroporation (electrodes location indicated with arrows), revealing the spatial pattern of current penetration within the targeted region (adapted from [15]).

Afterwards, in vivo animal experiments involving healthy beagles with induced brain abscess corroborated the potential of MR‐EIT to provide conductivity information of tissues in situ (Figure 3A) [13]. Clear conductivity contrast was observed between normal tissue and abscess lesions, where the conductivity rose until 12 h post‐injection—a change driven by inflammation and edema—then declined after 18 h as necrosis, hemorrhage, and cellular breakdown progressed. Histological examination supported these findings, revealing central necrosis and inflammation with peripheral edema and hemorrhage that explain the observed conductivity dynamics.

Recent research work also showed the potential of conductivity imaging to detect microstructural brain changes in patients with neurological diseases such as Alzheimer's disease. In a small cohort study, increased extra‐neurite conductivity in patients with Alzheimer's disease compared with healthy controls was observed [14], which is a finding consistent with neuronal loss. These preliminary results are promising but require confirmation in larger, well‐characterized cohorts to determine robustness, diagnostic accuracy, and how conductivity measures correlate with established clinical and biomarker indices.

Because tissue conductivity depends on temperature, rapid conductivity imaging methods have been developed to monitor spatial temperature distributions during radiofrequency (RF) ablation (Figure 3B) [17]. This study showed the capability of MR‐EIT to monitor temperature and structural changes in tissues during RF ablation by producing spatio‐temporal maps of tissue conductivity every 10 s. This may offer a possibility for improved treatment guidance and real‐time feedback to optimize ablation efficacy.

More recent studies showed the feasibility of using MR‐EIT to map electric field distributions during electroporation, providing real‐time assessment of reversible and irreversible electroporation zones in tumor models (Figure 3C) [15]. MR‐EIT accurately predicted the extent of reversibly electroporated tumor regions, correlating strongly with both gadolinium‐based contrast entrapment and histological staining, highlighting its value for optimizing electrochemotherapy and irreversible electroporation procedures.

## 3 High Frequencies Conductivity Imaging

### 3.1 Theory

At Larmor frequencies (RF, typically 64–298 MHz), tissue electrical properties perturb external electromagnetic fields as they propagate through the sample, such as the transmit magnetic field (*B*_1_^+^) induced by a transmit RF coil during an MRI scan. Unlike MR‐CDI or MR‐EIT, MR‐EPT leverages such interaction to retrieve the electrical properties of tissues from noninvasive measurements of the *B*_1_^+^ field, without needing current injections through external electrodes [85, 86, 87].

In its classical formulation, MR‐EPT relies on the differential form of Maxwell's equations and aims to reconstruct tissue electrical properties from measured *B*_1_^+^ maps. However, to make this inverse problem tractable, several assumptions are often made.

First, conductivity and permittivity are usually assumed isotropic.

Second, the magnetic permeability is typically assumed constant and equal to that of free space. While valid for biological tissues, this assumption may not hold in the presence of implants or contrast agents.

Third, electrical properties are assumed to vary slowly or be constant within a small region, that is, they are considered locally homogeneous: local homogeneity assumption. This simplifies the reconstruction formulation but fails near tissue boundaries or in heterogeneous regions. The influence of tissue interfaces can lead to significant errors in reconstructed maps, for examples in brain, breast, or abdomen.

Fourth, the phase of the *B*_1_^+^ is calculated as half the phase of the measurement, so‐called transceive phase (*φ* ^±^), which is the sum of the transmit (*φ* ^+^) and receive (*φ* ^−^) phases that are usually assumed to be equal. This is a valid approximation only when using certain transmit coils and it does not hold above certain frequencies (298 MHz and above).

Fifth, conductivity is assumed to affect only the phase of *B*_1_^+^, which enables reconstructions from phase‐only data. This holds for the central region of homogeneous and symmetric samples and at field strengths lower than 3 T (128 MHz).

### 3.2 Acquisitions

The full implementation of MR‐EPT involves measuring the complex RF transmit field component *B*_1_^+^, that is, its magnitude |*B*_1_^+^| and its phase *φ* ^+^, approximated via the above‐mentioned transceive phase assumption. Although sequences with simultaneous measurements of |*B*_1_^+^| and *φ* ^±^ are possible, the current practice is to measure these two quantities separately.

To obtain |*B*_1_^+^|, numerous *B*_1_‐mapping techniques can be used. More important than potential differences in accuracy among these techniques is the need for high SNR in the applied *B*_1_‐mapping method.

To obtain the transmit phase *φ* ^+^, transceive phase images can be taken from standard sequences if these images relate exclusively to RF penetration (typically SE based or balanced steady‐state free‐precession sequences). These *φ* ^±^ phase images are then divided by 2 to cope with the superposition of RF transmit and receive components.

For more details and corresponding references, we refer the reader to a separate, comprehensive guideline [88].

### 3.3 Reconstructions

Various physics‐based reconstruction algorithms have been developed in MR‐EPT to estimate tissue electrical properties. These physics‐based methods are grounded in different assumptions and practical considerations, aiming to reduce reconstruction errors and enhance clinical reliability. Most of the physics‐based reconstruction approaches are presented and discussed in depth in a methodological review paper [89].

For practical reasons and simplicity in acquisition and reconstructions, most MR‐EPT in vivo applications rely on the phase‐only reconstruction formulation, which requires measuring only the transceive phase. However, this formulation approximates derivatives of noisy measurements using finite differences, which leads to severe noise amplification and assumes that electrical properties are locally homogeneous, leading to severe errors at tissue interfaces [90, 91]. To address these challenges, several alternative reconstruction strategies have been proposed for clinical applications.

First, by relying on the local homogeneity assumption, conductivity reconstructions were performed by fitting the measured phase within regions exhibiting similar MR signal intensity with second order polynomials, under the assumption that these areas share homogeneous electrical properties. This method typically leverages MR image contrast to restrict the fitting kernel to voxels with comparable tissue intensities, thereby maintaining the piece‐wise constant property without necessitating explicit tissue segmentation. In contrast to these traditional Helmholtz‐based (phase‐only) techniques, another prominent approach reformulates the generalized Helmholtz equation into a convection‐reaction formulation [89], leading to the development of convection‐reaction EPT (cr‐EPT) [18, 92]. Unlike Helmholtz‐based methods, cr‐EPT does not assume tissue homogeneity or require explicit MR contrast guidance, thereby enabling more generalizable and flexible conductivity reconstruction across diverse tissue types. Furthermore, the integration of Savitzky–Golay filtering enables adaptive derivative kernel size selection, further improving resilience to noise in the reconstruction process [93].

In recent years, DL approaches have emerged as an alternative to physics‐based MR‐EPT algorithms, aiming to address their inherent limitations and improve reconstruction accuracy and efficiency [19, 20, 21, 22, 23, 24, 94, 95, 96, 97]. Various supervised learning models such as artificial neural networks (ANNs), convolutional neural networks (CNNs), and vision transformer‐based architectures have been applied to phase‐based conductivity reconstruction. Most DL‐based methods are implemented as direct end‐to‐end estimators, which require large and exhaustive training datasets to ensure sufficient generalization, and are trained on simulated data due to the lack of ground‐truth conductivity measurements in vivo [19, 20, 21]. This leads to lower performance when applied to in vivo data.

To mitigate these issues, recent research has explored hybrid models that integrate DL with established physical frameworks, leading to physics‐informed neural networks [23, 94, 95, 96, 97]. By embedding physical priors into the network architecture, these models enforce physical‐informed or ‐constrained reconstructions, thereby enhancing the reliability and interpretability of the results. Preliminary studies on patients further support their promise as a robust and generalizable solution for clinical application [23].

As briefly summarized, the evolution of MR‐EPT reconstruction, from early numerical formulations to more advanced techniques such as polynomial fitting, convection‐reaction models, and DL, demonstrates a continued effort to balance accuracy, robustness, and clinical applicability. Phase‐based EPT methods have become predominant in practice due to their simplified acquisition requirements. Nevertheless, their inherent limitations, including noise sensitivity and artifacts at tissue interfaces, continue to drive innovation in reconstruction methodologies. The advent of physics‐informed and physics‐constrained DL represents a significant paradigm shift, enabling synergistic integration of data‐driven models with physical principles. Moving forward, future research should aim to develop hybrid frameworks that effectively combine model‐based and learning‐based advantages and validate them extensively on in vivo data to facilitate clinical translation of MR‐EPT as a reliable tool for noninvasive tissue characterization.

### 3.4 Pre‐Clinical Applications

**FIGURE 4:**
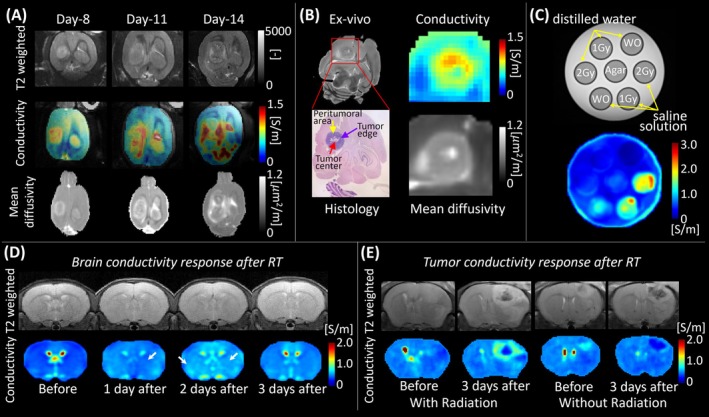
(A) Conductivity imaging of tumor growth after implantation in a mouse. Tumor progression is characterized by increasing conductivity and decreasing mean diffusivity, reflecting cellular proliferation and microstructural restriction (adapted from [26]). (B) Ex vivo tumor histological validation. Elevated conductivity at the viable tumor rim corresponds to regions of high cellularity confirmed histologically (adapted from [26]). (C–E) Conductivity changes in response to radiation in: (C) A phantom irradiated with 1–2 Gy demonstrating a dose‐dependent rise in conductivity (adapted from [28]). (D) Mouse brains after a single 10 Gy exposure up to 10 days post‐radiation (adapted from [28]). (E) Mouse brains with tumors revealing localized conductivity increase confined to the irradiated hemisphere after 5 Gy radiation versus no mouse brains without radiation (adapted from [29]).

Preclinical animal studies across neurological and systemic models demonstrated the sensitivity of MR‐EPT in detecting microenvironmental tissue changes, particularly for monitoring tumor growth, aggressiveness, and response to therapies such as radiation. In F98 glioblastoma‐bearing rats, high frequency conductivity progressively increased in the tumor rim, viable core, and surrounding edema over time, even when diffusion metrics like mean diffusivity remained stable (Figure 4A) [26]. These changes, validated by histology and high resolution ex vivo imaging, reflected increased cellularity and ionic concentration during tumor progression (Figure 4B) [26]. Similar findings were reported in breast and prostate tumor‐bearing rats, where tumor conductivity was significantly higher than surrounding tissue, consistent with dielectric probe measurements [51, 68].

Conductivity imaging was also applied to monitor radiation therapy (RT) response in multiple preclinical models [27]. In agar‐saline phantoms irradiated with 1–2 Gy, conductivity increased proportionally with dose, confirming the sensitivity of conductivity mapping to radiation‐induced ionic changes (Figure 4C) [27, 28]. In tumor‐bearing mouse brains exposed to 10 Gy using the X‐RAD SmART system, conductivity exhibited a clear time‐dependent rise over 1–10 days post‐irradiation, demonstrating higher sensitivity than T2‐weighted or diffusion MRI (Figure 4D) [28]. At 5 Gy, comparative imaging further distinguished irradiated from nonirradiated hemispheres, revealing localized conductivity elevation confined to the treated region [28]. Extending these findings, peri‐irradiation studies in brain tumor models showed significantly higher conductivity in irradiated tumors compared with controls, with percentage changes differing between tumor rims and cores, indicating that MR‐EPT can capture intratumoral heterogeneity and quantify therapeutic response (Figure 4E) [29].

Beyond RT applications, conductivity imaging showed promise in a hypoxic–ischemic brain injury (HIBI) model in minipigs, where cerebral conductivity increased significantly within 1‐h post‐cardiac arrest [35]. In rats, *N*‐methyl‐D‐aspartate‐induced seizure models, hippocampal conductivity declined within hours, correlating with neuronal loss [34]. In Sprague–Dawley rats injected with dimethylnitrosamine, fibrotic liver regions showed ≥ 12% higher conductivity, consistent with extracellular accumulation of proteins, collagen, and inflammation [65].

Collectively, these studies highlight the ability of conductivity imaging to noninvasively detect early and dynamic tissue changes in tumors, ischemia, fibrosis, seizures, and radiation injury well before structural alterations are visible on conventional MRI.

### 3.5 Clinical Applications

#### 3.5.1 Brain Tumors

The first clinical applications of MR‐EPT demonstrated its utility in distinguishing between different grades of diffuse glioma, the most common primary brain tumor in adults [30].

**FIGURE 5:**
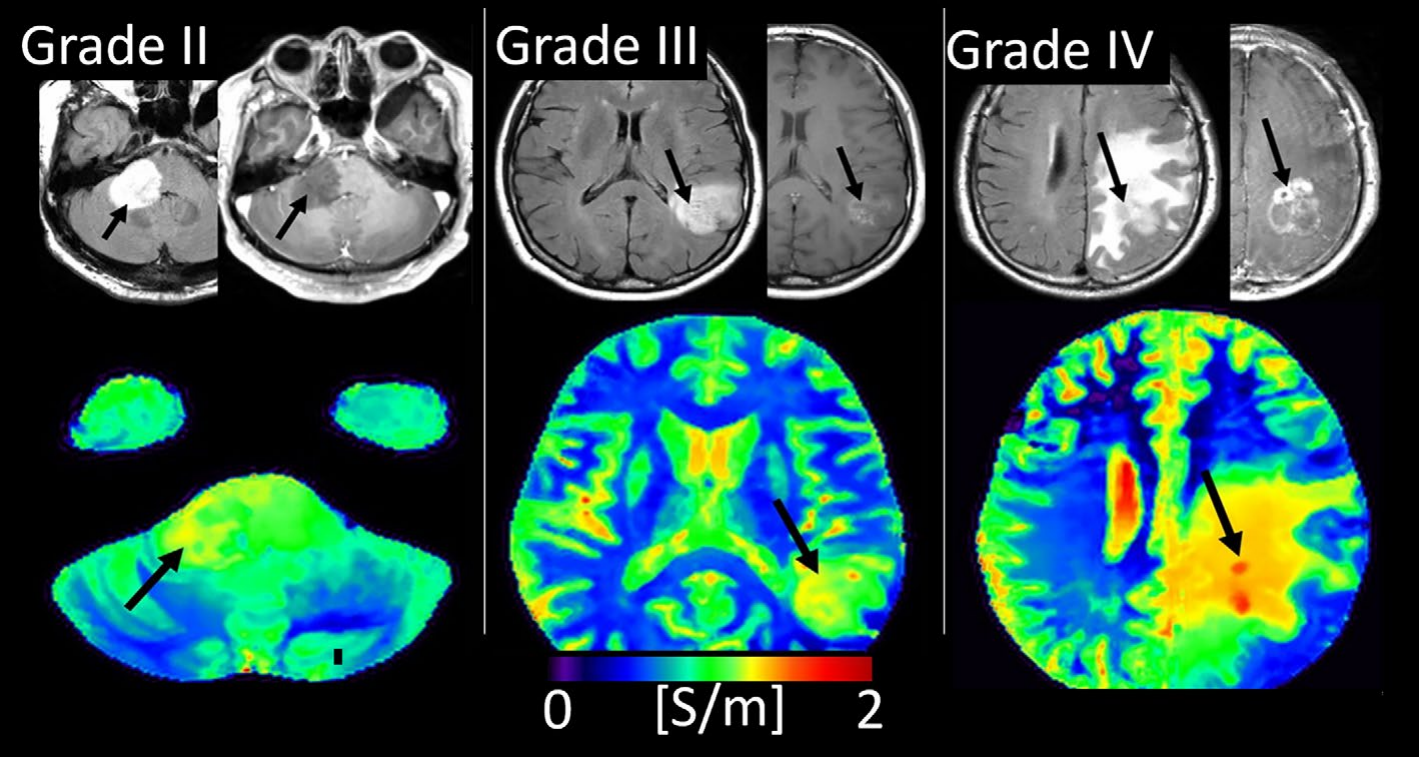
Conductivity maps of diffuse glioma. Grade II (left) to Grade IV (right) according to 2016 WHO classification of Central Nervous System tumors. The upper row shows axial FLAIR (left) and contrast‐enhanced T1‐weighted images (right), and the lower row shows the corresponding conductivity maps. Tumors are indicated by black arrows.

Higher grade gliomas exhibited higher conductivity than lower‐grade gliomas and healthy brain parenchyma (Figure 5), as also observed in conductivity maps derived from water‐content imaging [25]. Elevated tumor conductivity may arise from increased intracellular sodium in rapidly proliferating tumor cells, the influx of extracellular sodium due to a compromised blood–brain barrier, or myelin degradation. Additionally, the authors reported higher conductivity in contrast‐enhanced tumor regions compared to nonenhanced areas, highlighting the potential of conductivity as a predictive marker for contrast enhancement. These findings were further supported by similar observations in rat brain tumor models [26].

Beyond tumor grading, conductivity imaging showed potential for identifying microstructural tumor habitats. A longitudinal study in post‐treatment glioblastoma demonstrated that MR‐EPT could reveal a unique hypovascular low‐conductivity habitat that strongly predicted tumor progression when combined with cerebral blood volume (CBV) mapping [31]. This habitat outperformed diffusion‐ and perfusion‐based biomarkers and achieved an AUC of 0.86 for differentiating tumor progression from treatment‐related change, with combined multiparametric habitats reaching an AUC of 0.90. The study also reported that post‐treatment changes in glioblastoma were associated with higher conductivity than true tumor progression, likely reflecting differences in cellular membrane disruption and water content following chemoradiotherapy.

**FIGURE 6:**
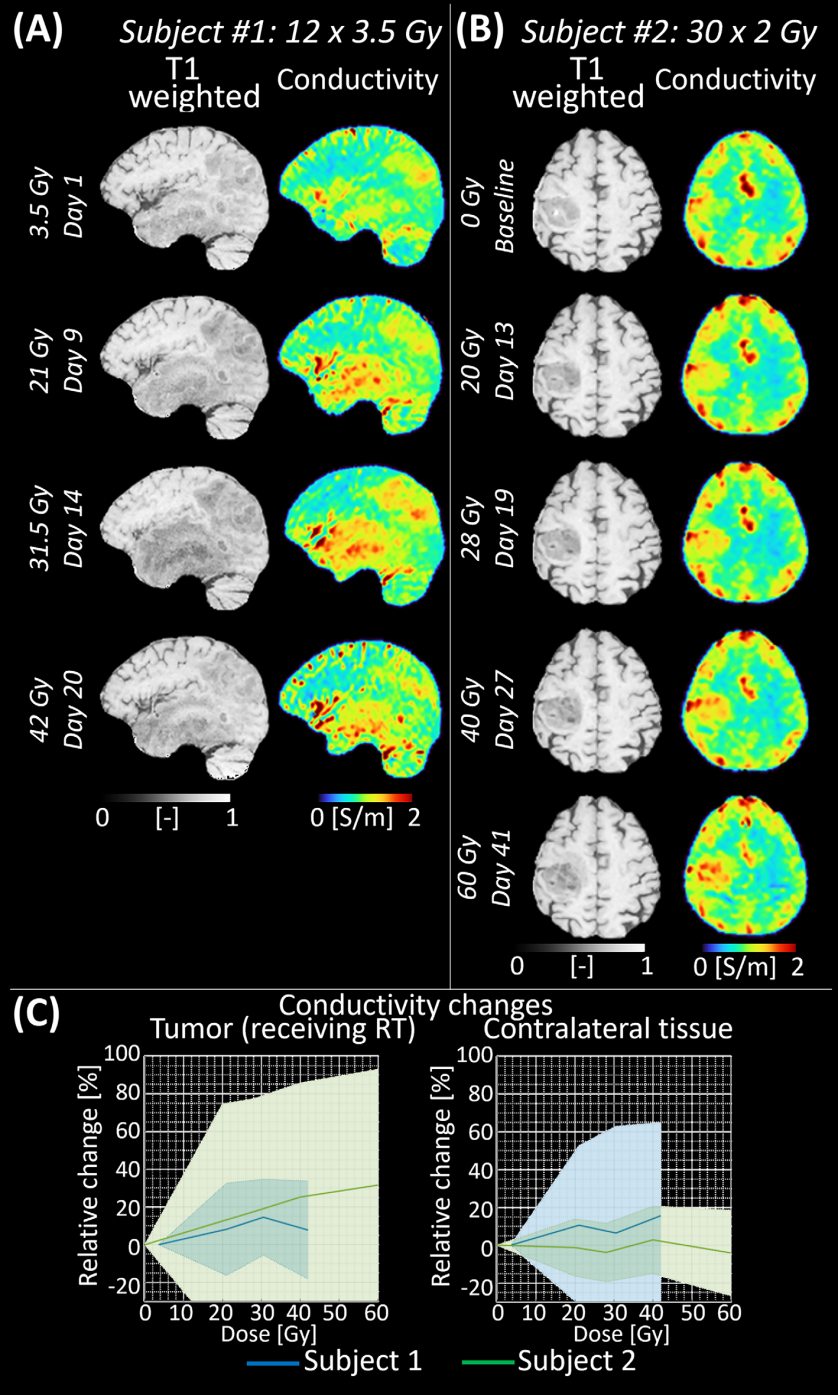
Example of conductivity changes during radiotherapy for two glioblastoma grade IV, IDH‐wildtype. (A, B) Qualitative images are shown for each MRI during the weeks of radiotherapy (RT). (C) Relative percentage difference within the tumor and contralateral tissue, obtained by mirroring the tumor mask and used as a reference for each time point with respect to baseline. *Note*: Subject #1 shows a rapid increase in conductivity at Day 14 caused by radiation‐induced edema, resolved after dexamethasone administration. Conductivity changes remained < 15%. Instead, Subject #2 showed a steady increase in conductivity leading to about +35%. Such changes may serve as early indicators of tumor responsiveness, reflecting changes in tissue cellularity, water, ionic and protein content, and may be used for treatment response monitoring and early clinical‐decision making.

Building on pre‐clinical studies that showed conductivity changes in response to RT preceding structural changes [27, 29], conductivity imaging was recently acquired in two glioblastoma patients during the weeks of RT [32]. In one patient, who died soon after RT, no clear conductivity changes were observed in the tumor. Conversely, in the other patient, alive 7 months post therapy, an increase in tumor conductivity over time was observed, leading to > 30% conductivity increase at the end of treatment (Figure 6). These preliminary results suggested that conductivity imaging may serve as a valuable tool for early treatment response and longitudinal treatment monitoring.

Despite these promising findings, the clinical applicability of conductivity imaging in other brain tumors remains to be established, largely due to the scarcity of reports, which may be related to the relatively low prevalence of these tumors. An initial study showed higher conductivity in meningiomas compared to the surrounding brain parenchyma, and this increase in conductivity appears to be independent of tumor grade [33]. It may be worth further exploring whether conductivity can play a role in distinguishing various histological types of meningioma that vary in the degree of vascularity, cystic formation, and calcification.

#### 3.5.2 Brain Stroke

Brain conductivity changes after stroke depend on stroke type (ischemic or hemorrhagic) and the phase post‐stroke.

In ischemic stroke, energy failure, ionic imbalance, and cytotoxic edema initially reduce conductivity in the ischemic core. Afterwards, conductivity may rise due to the release of intracellular contents from necrotic cells, while in the chronic phase conductivity varies with tissue loss, gliotic scar formation, and residual edema. In the penumbra, acute changes are complex, possibly showing an initial decrease due to cellular swelling followed by a possible increase caused by edema. In the subacute phase, conductivity reflects vasogenic edema and ongoing cellular damage, whereas, in the chronic phase, conductivity changes depend on tissue recovery or gliosis.

In hemorrhagic stroke, the hematoma core shows increased conductivity in the acute phase due to blood release. Subacutely, conductivity within the hematoma may decrease as clotting occurs, though surrounding edema may show increased conductivity. In the chronic phase, conductivity depends on the hematoma resolution, hemosiderin deposition, and residual tissue damage. In the perihematoma region, acute changes are milder but influenced by edema and blood–brain barrier disruption, while later phases reflect edema resolution and effects of blood‐derived products.

**FIGURE 7:**
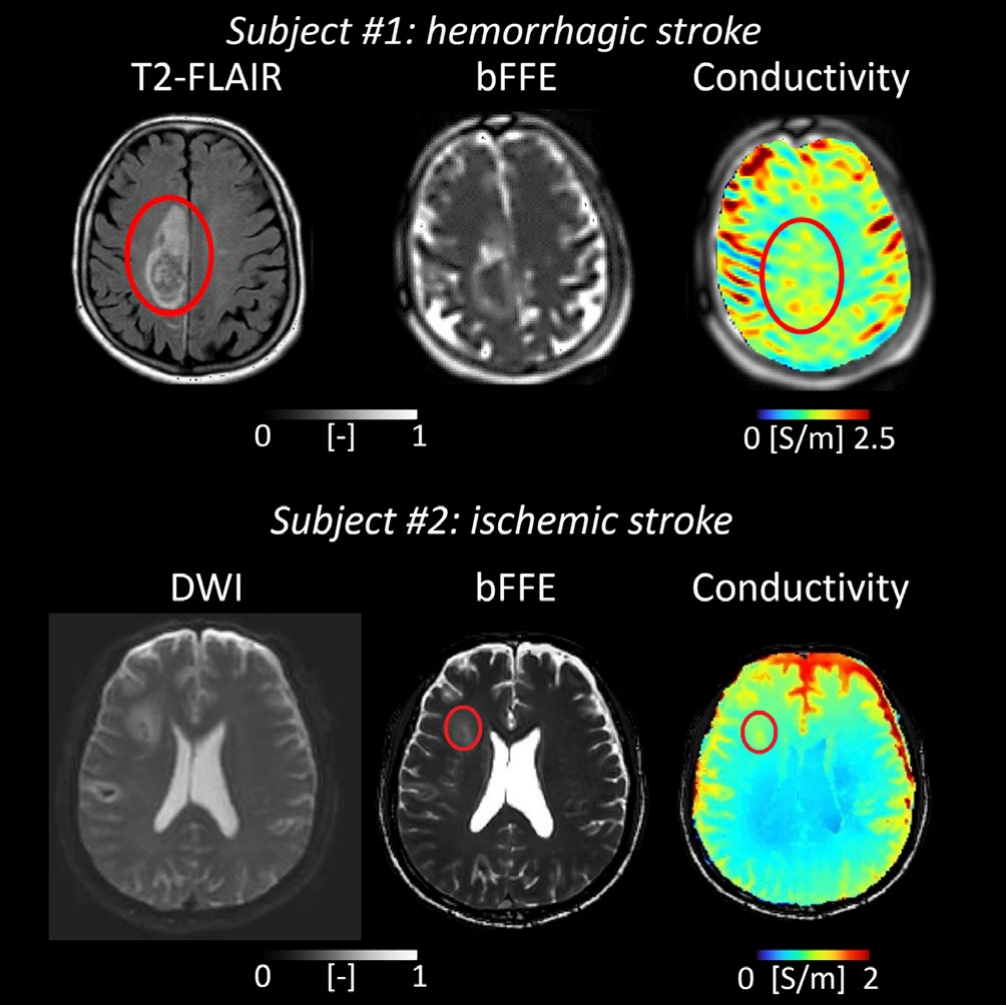
Examples of hemorrhagic (hematoma and surrounding edema) and ischemic stroke showing higher conductivity values (red circles) (adapted from [37, 38]).

A 7 T feasibility study on an ischemic stroke patient 2 months post‐stroke reported twofold higher conductivity in the infarcted region, reflecting ionic imbalance, cytotoxic edema, and membrane breakdown [36]. Another study using MR‐EPT in hemorrhagic and ischemic stroke reported increased conductivity in ischemic lesions and perihematomal edema, but not within hematomas, likely due to clot formation (Figure 7, top) [37]. More recently, MR‐EPT in ischemic stroke patients ~100 h post‐stroke showed significantly higher conductivity in lesions in the basal ganglia, thalamus, and cerebral hemisphere compared to contralateral tissue (Figure 7, bottom) [38]. Finally, a recent MR‐EPT study in stroke patients undergoing autologous stem‐cell transplantation found regional conductivity increases across the disease course [39]. Conductivity at the precentral gyrus (2 weeks post‐infarction, before transplantation) correlated positively with 1 year modified Rankin scores, underscoring MR‐EPT's potential for therapy monitoring. Together, these studies demonstrate MR‐EPT's sensitivity to ionic and structural variations in different stroke subtypes and establish MR‐EPT as a promising tool for characterizing infarct evolution, differentiating ischemic and hemorrhagic lesions, and evaluating regenerative interventions.

#### 3.5.3 Brain Degeneration and Demyelination

**FIGURE 8:**
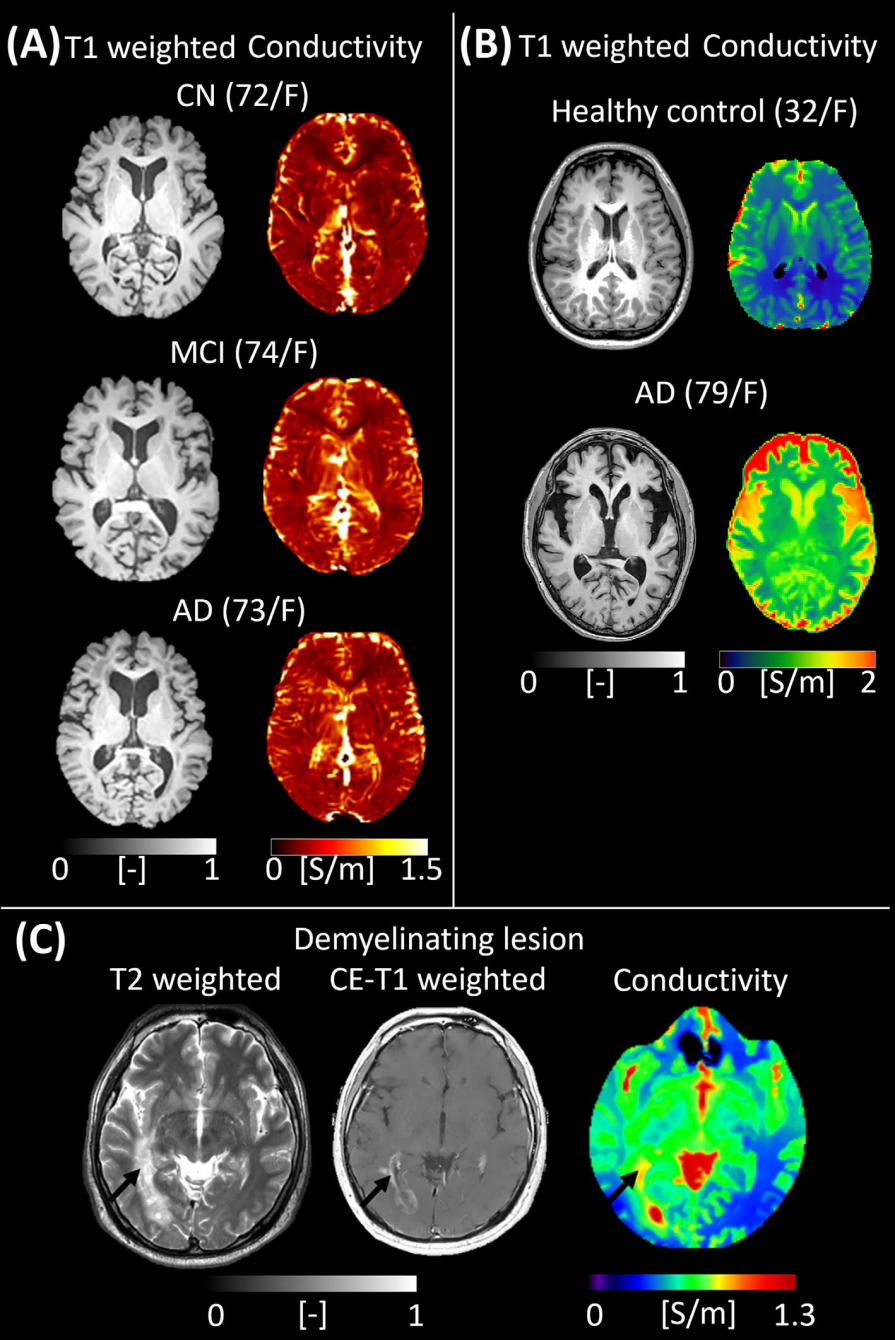
Representative brain conductivity maps across cognitive stages and pathological conditions. (A) T1 weighted and conductivity maps for a cognitively normal (CN), mild cognitive impairment (MCI), and Alzheimer's disease (AD) subject. A gradual elevation of conductivity is observed from CN to MCI to AD, most prominently in the temporal and parietal cortices and hippocampal regions (adapted from [40]). (B) Examples of conductivity maps from a normal control, a patient with AD that shows diffusely elevated conductivity across cortical and subcortical regions, consistent with tissue alterations associated with neurodegeneration. (C) T2 weighted (showing heterogeneous hyperintensity), contrast‐enhanced T1 weighted (demonstrating incomplete ring‐like enhancement), and conductivity maps (showing focally increased conductivity within the lesion—black arrows) for a patient with a demyelinating lesion.

Recent studies showed the potential of conductivity imaging for assessing neurodegenerative and demyelinating conditions, where conventional MRI often falls short in reflecting the underlying biochemical and microstructural alterations. In Alzheimer's disease, widespread elevation of conductivity in the brain parenchyma of AD patients, compared to those with mild cognitive impairment (MCI) and cognitively normal (CN) controls, was observed (Figure 8A) [40]. The observed increase in conductivity was attributed to the expansion of the extracellular space, as confirmed by concurrent DWI. More importantly, conductivity values correlated negatively with Mini‐Mental State Examination (MMSE) scores, suggesting that conductivity imaging may serve as a surrogate marker of cognitive decline. The study further demonstrated that combining insular high frequency conductivity values with hippocampal gray matter volume enhanced the classification performance for distinguishing AD from CN subjects, underscoring the complementary role of EPT in a multiparametric imaging framework (Figure 8B).

While reports on the application of conductivity imaging in other neurodegenerative diseases are lacking, increased regional conductivity values, due to expanded extracellular fluid volume and myelin breakdown, are expected. In a recent report, it was shown that a tumefactive demyelinating lesion, often challenging to differentiate from glioblastoma, exhibited slightly elevated conductivity compared to the surrounding white matter (Figure 8C) [41]. The magnitude of conductivity increase was less pronounced than that observed in glioblastoma. This subtle difference suggests that EPT may aid in differential diagnosis when conventional imaging findings are equivocal. In addition, an experimental study involving animal spinal cord reported sensitivity of EPT in identifying spinal cord regions with diverse myelin distribution [70]. Collectively, these studies underscore the value of conductivity imaging not only in detecting and localizing pathology but also in offering biophysical insights into disease mechanisms.

#### 3.5.4 Brain Infection and Inflammation

Infections and inflammatory processes within the central nervous system (CNS) provoke complex tissue alterations, including edema, cellular infiltration, and increased protein concentration in CSF. These pathophysiological changes can affect the electrical properties of affected tissues.

**FIGURE 9:**
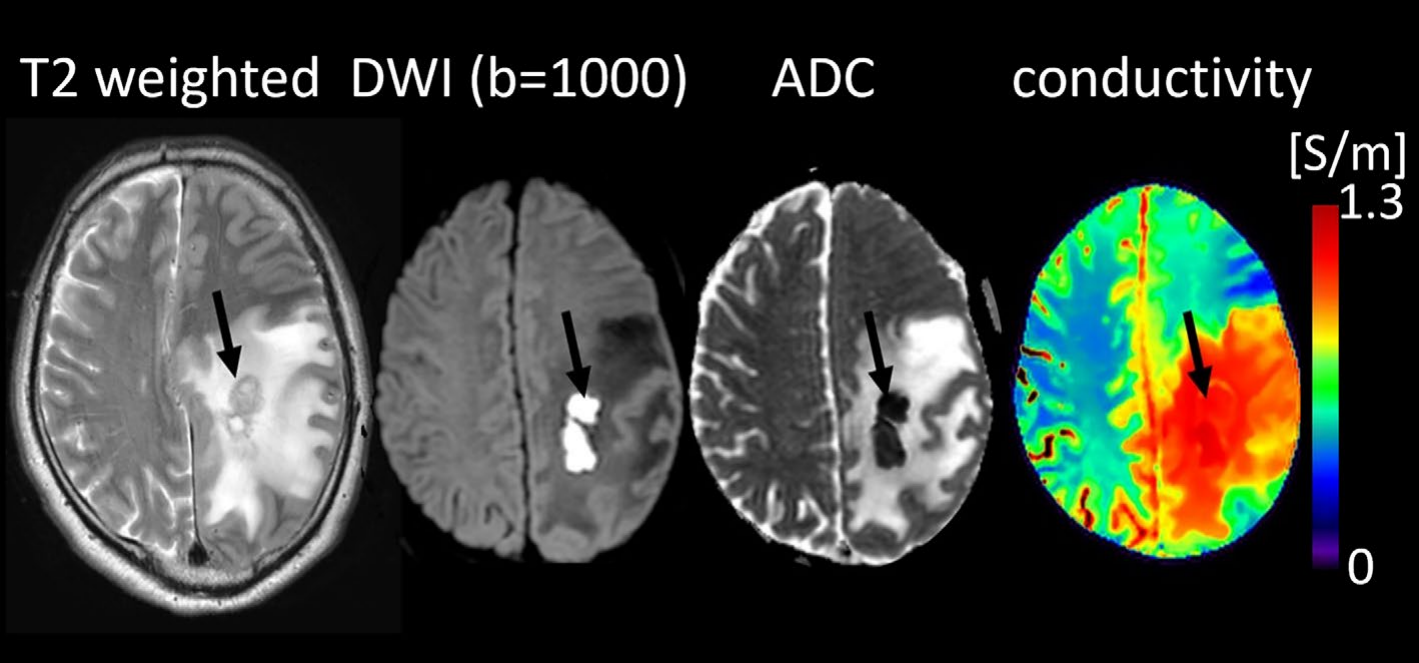
A case of cerebral abscess. T2 weighted image shows two ring‐like lesions (black arrows) and surrounding edema. DWI (*b* = 1000 s/mm^−2^) shows hyperintensity and the corresponding ADC map shows low ADC values, within the lesions—suggestive of restricted diffusion. Conductivity map shows very high conductivity within the lesions and surrounding edema.

While direct human studies applying MR‐EPT to infectious conditions remain scarce, the first report in a patient with cerebral abscess showed increased conductivity values compared to surrounding brain parenchyma (Figure 9) [41]. Despite technical challenges, CSF conductivity analyses showed a moderate positive correlation of conductivity histogram metrics with the CSF albumin concentration and total cell count—the common findings in CNS infection and inflammation [42]. Further studies are warranted to validate these initial observations. The development of a noninvasive yet sensitive technique to diagnose or monitor CNS infection and inflammation is especially important, as most current techniques are more or less invasive and conventional MRI sequences are not sensitive enough to detect trivial alterations in CSF biochemical composition.

#### 3.5.5 Breast Tumors

**FIGURE 10:**
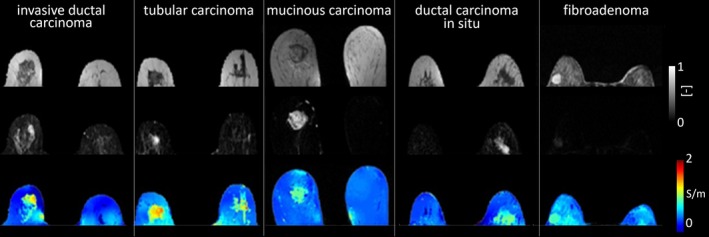
Breast tumor images from five patients, each diagnosed with a different condition. Top row: magnitude images; Middle row: Dynamic contrast enhanced images; Bottom row: conductivity images (adapted from [50]).

Breast conductivity imaging has demonstrated potential in tumor detection, characterization, and prognostication, particularly through noninvasive, phase‐based MR‐EPT approaches (Figure 10) [50, 52]. This interest arises from both the biological heterogeneity of breast tissue, comprising fibroglandular, adipose, and ductal elements, and the increasing clinical need for noncontrast imaging alternatives, prompted by concerns over gadolinium‐based contrast agents.

The first in vivo clinical study demonstrated that malignant breast tumors exhibit significantly higher conductivity (mean ≈ 0.89 S/m) than benign lesions (0.56 S/m) and normal parenchyma (0.43 S/m) [50]. These findings were expanded through correlations between conductivity and prognostic markers, including human epidermal growth factor receptor‐2 (HER‐2) status, which is related to tissue aggressiveness, and mitotic index [53]. In addition, conductivity showed a moderate inverse correlation with apparent diffusion coefficient (ADC) in nonnecrotic tumor [54], and comparable diagnostic performance to contrast‐enhanced MRI measures such as the signal enhancement ratio (AUC: 0.71 vs. 0.80) [55]. Although cancer detection sensitivity using conductivity imaging is lower than conventional modalities (20%–32% vs. 62%–90%), its quantitative metrics, particularly maximum conductivity, showed moderate discriminatory power (AUC: 0.65–0.67), especially in fatty breast tissue or larger tumors [52]. From a prognostic perspective, mean conductivity showed a low but significant correlation with FDG‐PET standardized uptake peak value (*r* = 0.381), and was higher in tumors with lymphovascular invasion, suggesting its potential as a noninvasive marker of tumor aggressiveness [56]. Moreover, in a prospective study targeting reduction of unnecessary biopsies for Breast Imaging Reporting and Data System (BI‐RADS) 4 lesions, conductivity imaging decreased false‐positive biopsy rates by 23% without missing malignancies, offering a contrast‐free alternative to DWI (38%) and abbreviated MRI (43%) [57].

Breast conductivity imaging therefore represents a developing yet promising application of MR‐EPT. While further validation is needed to address spatial resolution, chemical shift effect, and standardization, current evidence suggests that conductivity imaging may complement conventional imaging by offering contrast‐agent‐free insights into tumor biology, aiding diagnosis and prognosis.

#### 3.5.6 Thoracic Lesions

MR‐EPT, like other MRI sequences, faces challenges in thoracic applications due to low proton content and very short T2 components in lung tissue, abrupt magnetic susceptibility variations at tissue interfaces, and cardiothoracic motion. However, a few recent studies suggested its potential in evaluating lung and mediastinal lesions. Measurements based on ultra‐short or zero echo‐time provided phase maps suitable for conductivity reconstructions, offering an advanced option for investigating conductivity of the lung. A preliminary study on healthy volunteers demonstrated conductivity differences in the lungs between inspiration and expiration [58].

**FIGURE 11:**
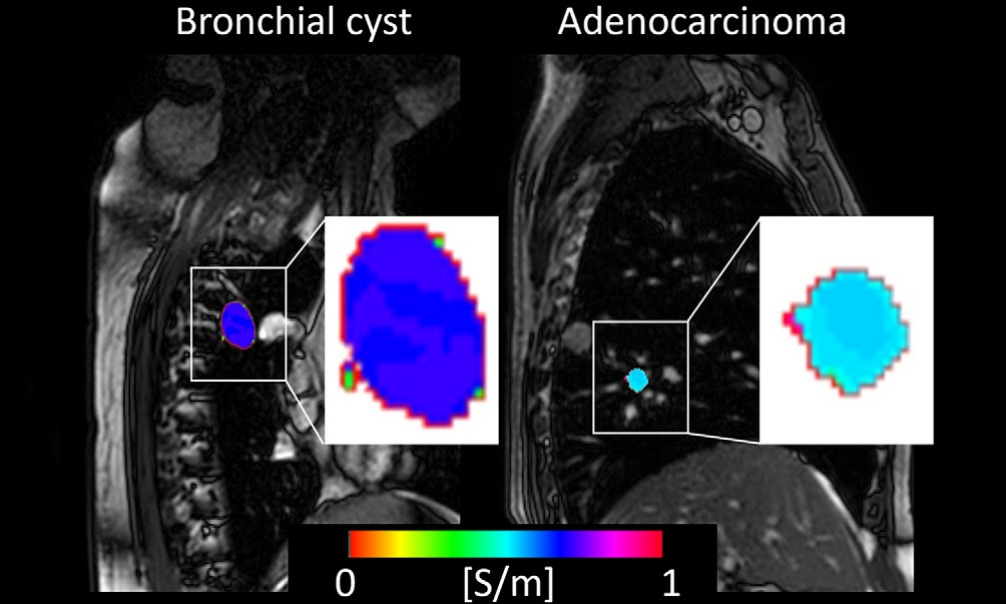
Conductivity of bronchial cyst (left) and adenocarcinoma (right). Reconstructed conductivity of the segmented lesions is overlaid on the corresponding magnitude images of 2D‐SSFP. Magnified conductivity maps of the segmented lesions are also given (white background).

More recently, a preliminary study applied conductivity imaging at 1.5 T in patients with lung and mediastinal lesions [59]. Lesion histogram and texture analyses revealed significantly higher contrast features in malignant lesions, indicating greater intratumor heterogeneity (Figure 11). No significant difference in conductivity distribution was observed between upper and lower lung lesions, suggesting minimal impact from cardiothoracic motion. A subsequent study demonstrated that the diagnostic performance of conductivity in distinguishing benign from malignant thoracic lesions is comparable to that of ADC [60]. This finding may shed light on the potential role of MRI‐based conductivity in evaluating lung lesions and respiratory diseases.

#### 3.5.7 Heart Muscle and Blood

Myocardial function is associated with active electrophysiology. The concerted excitation across the myocardium as well as the contraction of the cardiomyocytes is realized by actively driven ionic processes. Comprehensive cardiac electrophysiology, however, includes not only active but also passive electrophysiologic aspects, particularly ionic concentration and mobility. Unfortunately, measurement of cardiac “bulk” conductivity via MR‐EPT is severely hampered by cardiac motion, as on one hand the measured *B*_1_ phase is extremely sensitive to motion, and on the other hand, derivation of conductivity from *B*_1_ phase is extremely sensitive to phase perturbations. These circumstances might account for the low number of corresponding studies published yet [61, 62, 63]. Following an initial study in two isolated, perfused pig hearts [61], two recent studies investigated myocardial and blood conductivity in healthy volunteers [62, 63]. Blood conductivity showed a reproducible pattern over the cardiac cycle with reasonable values during the cardiac resting phase [62]. Myocardial conductivity was reconstructed for the time point of largest myocardial extent, as reconstruction reliability increases with the number of voxels available for the tissue type of interest. While this study was based on *B*_1_ phase only [62], *B*_1_ magnitude was additionally used in [63]. The observed consistency in myocardial conductivity values between the two studies and existing literature highlights the potential of conductivity imaging as a noninvasive method for mapping myocardial conductivity, offering an indirect assessment of cardiac electrical activity, which may be altered in the presence of cardiac diseases like arrhythmias.

#### 3.5.8 Liver

**FIGURE 12:**
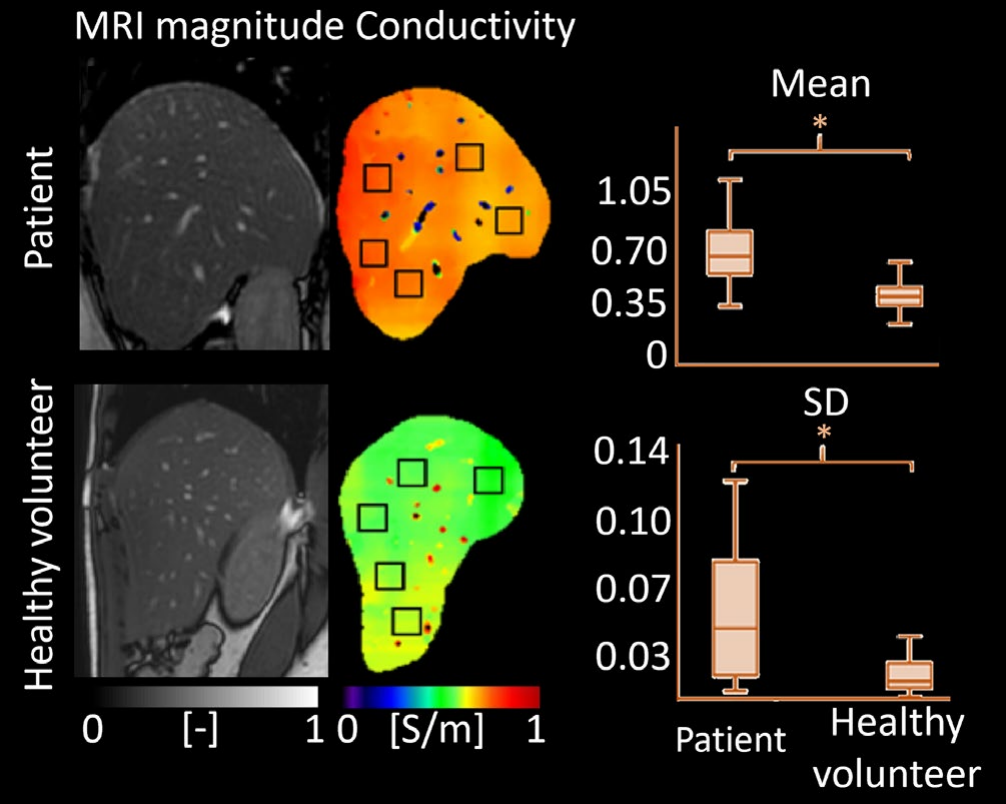
MRI bFFE magnitude images and conductivity maps of a healthy volunteer and a patient with hepatic cirrhosis (Stage F4). Conductivity of the liver parenchyma of the patient is much higher than that of the volunteer and shows much more variability (standard deviation—SD) in selected regions of interest (black squares), *p < 0.001 (adapted from [64]).

Conductivity has emerged as a promising biomarker for assessing hepatic fibrosis and cirrhosis. Initial evidence from experimental rat models using MR‐EIT demonstrated increased conductivity in fibrotic liver tissue [65]. These findings were later validated in a human study in 10 patients with confirmed hepatic fibrosis or cirrhosis and six healthy volunteers [64]. Conductivity images were acquired using a 1.5 T MRI system in sagittal sections of the liver parenchyma, avoiding focal lesions and vascular structures. Histogram‐based analysis of conductivity metrics revealed significantly higher mean, mode, minimum, maximum, median, and standard deviation values in patients with fibrosis or cirrhosis (Figure 12) [64], reinforcing MR‐EIT findings and suggesting frequency‐independent conductivity changes in liver disease. Conductivity elevation in hepatic fibrosis or cirrhosis was attributed to tissue changes in the extracellular space, primarily characterized by the excessive accumulation of extracellular matrix proteins, including collagen.

#### 3.5.9 Cervical Tumors

In a recent study, conductivity was measured in cervical cancer patients at 3 T [66]. It was observed that the mean value of all cervical tumors was ~1 S/m, which is 13% higher than ex vivo literature values, the mean value of muscle conductivity was ~0.93 S/m, which is 14% higher than literature values, and the mean values of bladder content was ~1.76 S/m, which is one order of magnitude higher than the value often used for bladder volume in numerical human models. In a subsequent study focused on hyperthermia treatments, it was observed that these differences have a substantial impact on cervical tumor temperatures achieved during hyperthermia [67]: a higher conductivity in the bladder and muscle tissue surrounding the tumor leads to higher power dissipation in the bladder and muscle, and therefore to lower tumor temperatures. These findings suggest the importance of using in vivo, and potentially subject‐specific, conductivity values for guidance of hyperthermia treatments.

#### 3.5.10 Musculoskeletal System

**FIGURE 13:**
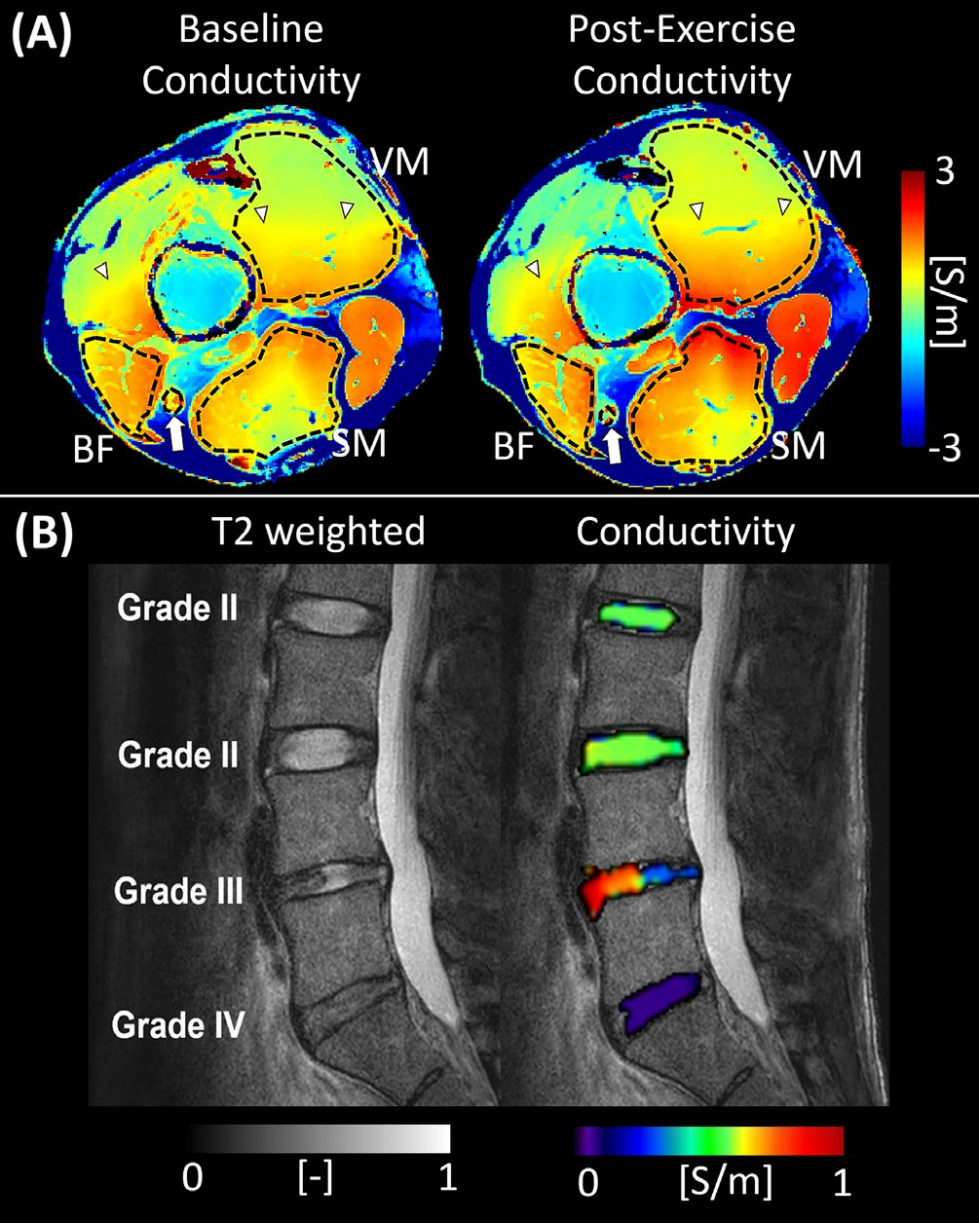
Conductivity imaging of musculoskeletal structures at 3 T. (A) Skeletal muscle conductivity before and after exercise (healthy subject) demonstrates regional increases most prominently in the vastus medialis (VM), semimembranosus (SM), and biceps femoris (BF) muscles, reflecting exercise‐induced perfusion and ionic concentration changes (adapted from [69]). (B) Sagittal lumbar spine conductivity grading. Reconstructed conductivity maps of lumbar intervertebral discs in a patient with intervertebral disc degeneration, overlaid on the SSFP magnitude image (right). The corresponding sagittal T2‐weighted image is shown on the left. As the Pfirrmann grade increases, indicating more advanced degeneration, conductivity decreases, reflecting reduced hydration and matrix integrity within the nucleus pulposus (adapted from [72]).

Emerging studies also indicated promising applications of conductivity imaging within the musculoskeletal system. In healthy volunteers, changes in muscle conductivity measured before and after exercise have been reported (Figure 13A) [69]. The change in conductivity is thought to reflect dynamic redistribution of sodium ions and water within muscles during physical activity, suggesting that EPT can sensitively capture physiological changes in muscle ion homeostasis and hydration status.

Beyond muscle, conductivity imaging has been applied to lumbar intervertebral discs, where it showed sensitivity to diurnal variations in disc hydration [71]. The well‐known daily fluctuation in water content, influenced by mechanical loading and unloading, results in measurable changes in conductivity, confirming MR‐EPT's ability to detect subtle biophysical alterations in discs. Furthermore, studies have extended this approach to degenerative disc disease, where pilot data indicate that EPT can distinguish between healthy and degenerated discs based on their conductivity values (Figure 13B) [72]. Importantly, preliminary clinical work suggests that conductivity imaging may also be used to monitor treatment response, showing conductivity differences between therapeutic regimens [73].

These initial findings highlight the potential of conductivity imaging as a noninvasive tool for musculoskeletal system assessment, particularly in disorders characterized by altered ion concentration and hydration, such as muscle fatigue and intervertebral disc degeneration. Further research is needed to validate and standardize these applications for clinical use.

### 3.6 Emerging Application: Functional EPT

**FIGURE 14:**
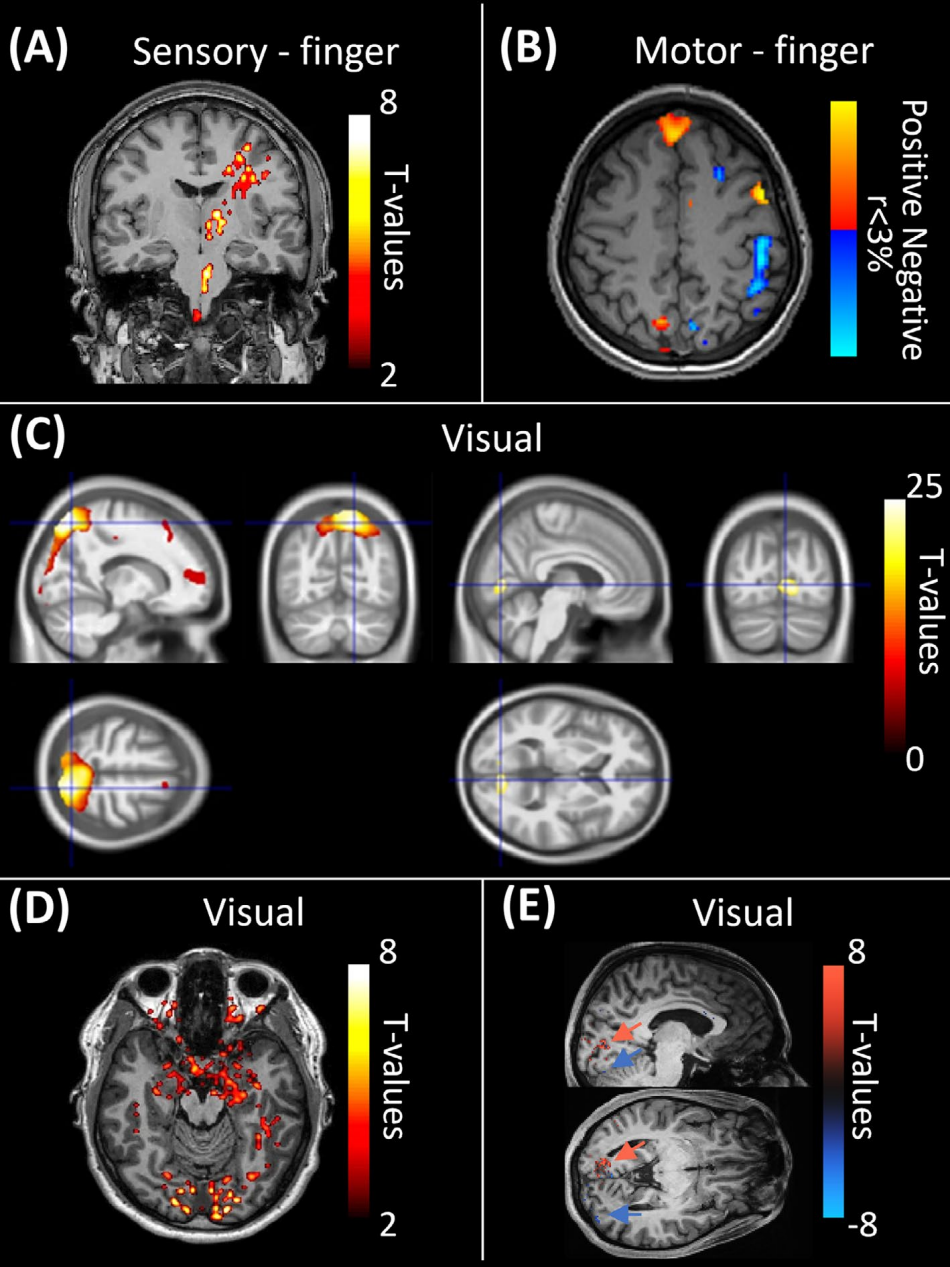
Sensorimotor task‐associated functional conductivity changes in different fEPT studies. (A) Conductivity increase correlated with scraping the right index finger in an event‐related study using 3D bFFE (adapted from [45]). (B) Conductivity decrease associated with finger‐tapping in 2D bSSFP fEPT using 192 averages of a single slice [48]. (C) Conductivity increases with visual stimulation in block‐design fEPT using 2D SE‐EPI (left) and 3D bFFE (right) (adapted from [46]). (D) Conductivity increase with visual stimulation observed in an event‐related study using 3D bFFE (adapted from [45]). (E) Conductivity changes with block‐design visual stimulation in fEPT using 2D ME‐GRE‐EPI. All activations were insignificant after removing contributions to EPT from BOLD magnitude signal changes [47].

MR‐EPT has recently been applied to functional neuroimaging [43, 44, 45, 46, 47, 48] (Figure 14). This approach, termed functional electrical properties tomography (fEPT), aims to reveal dynamic tissue electrical conductivity changes associated with neuronal activation.

#### 3.6.1 Acquisitions

fEPT requires high temporal resolution (volume TR ~2–4 s) and accurate *B*_1_^+^ phase acquisition. So far, fEPT studies, all at 3 T, have employed SE‐EPI [44, 46, 48] and bSSFP [43, 45, 46, 48], in 2D [44, 46, 47, 48], with single‐slice bSSFP signal averaging to improve SNR [48], and 3D [43, 45]. Some have used multiband acceleration [47] or compressed sensing [45]. SE‐EPI is rapid but suffers from geometric distortions, while bSSFP provides stable *B*_1_ phase but introduces banding artifacts. Multi‐echo gradient‐echo‐EPI (ME‐EPI) enables direct comparison between fEPT and simultaneously acquired standard fMRI [47]. Most studies have followed typical block paradigms with sensorimotor tasks, although a recent study has estimated a conductivity response function using an event‐related design [45].

#### 3.6.2 Reconstruction and Functional Analysis

All fEPT studies so far have used phase‐based EPT with various reconstruction algorithms. First‐order polynomial correction of eddy‐current effects can improve *B*_1_ phase stability [44, 48]. Although the effect of EPT reconstruction algorithms on fEPT is unclear, preliminary comparisons suggest that fEPT can be sensitive to these [48, 49].

Most fEPT studies have used a standard general linear model (GLM) approach, assuming a hemodynamic response function, although a different response function was measured in an event‐related fEPT study (Figure 14A) [45]. Correlation‐based analysis may more reliably detect fEPT activations than GLM under low‐SNR conditions (Figure 14B) [48].

#### 3.6.3 Challenges and Future Directions

In simulations and in vivo, *B*_1_^+^ phase increased on activation, with activated regions not always overlapping with conductivity activations [43, 48]. Nevertheless, observed functional conductivity changes are inconsistent (Figure 14) with both increases [43, 44, 45, 46, 47] and decreases [47, 48] reported, and positive conductivity activations seen in white matter [45].

Interpreting these discrepant fEPT results remains a challenge. Further challenges include fEPT's high noise‐sensitivity, highlighted by FDTD simulations [48], and lack of validation or understanding of relationships between divergent phase, conductivity, hemodynamic and neuronal changes. Optimizing acquisitions for high SNR and using denoising techniques, for example, deep‐learning [98] or MP‐PCA [99], may improve fEPT reliability. There is a need to systematically investigate the effects of different acquisition sequences, and reconstruction algorithms, and their respective parameters, on fEPT. Simulations and physical phantoms could be used to evaluate the spatial and temporal detection limits of fEPT, which is needed to establish it as a quantitative functional measurement. Multi‐modal imaging, integrating fEPT with MR‐EIT and other electrophysiological and functional imaging techniques, may enable further validation of fEPT and provide insight into underlying biophysical mechanisms, with potential to distinguish between intracellular and extracellular contributions. Addressing all these challenges would make fEPT a more accurate, reproducible, and clinically applicable tool for studying brain functions.

## 4 Discussion

As detailed above, conductivity imaging provides a unique window into the electrical properties of biological tissues. Unlike conventional MRI parameters that primarily reflect proton density or relaxation times, conductivity represents the ability of tissues to conduct electric current. This fundamental distinction offers complementary information that enhances both diagnostic, therapy, and research applications.

As highlighted in this review, tissue conductivity can be measured at different frequency ranges. While there is not a frequency range which is more valuable than the other, measurements at different frequencies require different techniques. For a detailed comparison between techniques, we refer the reader to a separate publication [100]. Additionally, at different frequencies tissue conductivity reflects different physiological features, such as ionic concentration, membrane integrity, and microstructural organization, making it sensitive to physiological and pathological alterations that may not be captured by standard MRI. For example, in neurological disorders, regional variations in brain conductivity may indicate microstructural disorganization, demyelination, or neuroinflammation, providing insights into disease mechanisms beyond those obtained from structural MRI or DWI.

Clinically, incorporating conductivity imaging into multiparametric MRI protocols enables more comprehensive tissue characterization. When combined with diffusion, quantitative relaxation, or magnetization transfer imaging, it enriches the evaluation of microstructural integrity and physiological states. Such integrative approaches may yield composite biomarkers that better differentiate disease stages, treatment responses, or prognostic subgroups. For example, in oncology, conductivity mapping serves as a surrogate indicator of ionic and microstructural alterations related to tumor growth, necrosis, and potentially response to therapy like chemoradiation. Malignant lesions often show elevated ionic content and membrane disruption, leading to altered conductivity compared with surrounding tissues. While conductivity is not a direct measure of tumor cellularity, it reflects the underlying ionic and structural milieu that frequently correlates with tumor aggressiveness and viability. This contrast mechanism complements functional and metabolic imaging methods, potentially improving lesion characterization, grading, and treatment monitoring.

From a translational standpoint, conductivity imaging also supports therapeutic optimization. In neurostimulation, transcranial current stimulation, and RF‐based interventions, subject‐specific conductivity maps improve electric field modeling, enabling individualized dosing and enhanced safety. Similarly, in cardiac and musculoskeletal imaging, conductivity information can assist in evaluating tissue viability, fibrosis, or edema.

Yet, despite its promising potential, conductivity imaging remains primarily a research tool, and several critical steps are necessary for its broader clinical adoption.

First, technical refinement is crucial for enhancing image quality, spatial resolution, and reproducibility. Developing standardized acquisition protocols and robust post‐processing algorithms will be crucial to ensuring reliable and reproducible measurements across various MRI platforms and clinical settings.

In this direction, efforts have been made by subgroups within the Electro‐Magnetic Tissue Property Study Group of the International Society for Magnetic Resonance in Medicine in defining three guidelines, respectively for MRI acquisitions for conductivity imaging [88], phantom preparations for testing conductivity reconstruction methods in controlled settings [101], and standardization of reporting results, including visualization of conductivity maps, data, and code sharing [102]. These are necessary to facilitate future development and objective comparison across novel conductivity reconstruction methodologies.

Second, large‐scale clinical studies are needed to validate conductivity imaging biomarkers against established diagnostic criteria and clinical outcomes. Multi‐center trials with diverse patient populations will help define normative values, pathological thresholds, and prognostic significance, facilitating regulatory approval and clinical acceptance. Integration with existing imaging workflows should be optimized to minimize additional scan time and complexity.

Third, interdisciplinary collaboration among physicists, radiologists, and clinicians will accelerate translational research and clinical validation. Education and training programs are essential for familiarizing healthcare professionals with the interpretation and application of conductivity maps.

Finally, exploring multimodal imaging approaches that combine conductivity imaging with diffusion, perfusion, and metabolic imaging will enhance diagnostic power and clinical relevance. As these advances mature, conductivity imaging is poised to transition from experimental research to a valuable clinical tool, improving diagnosis, treatment planning, and monitoring in various diseases.

In summary, these first clinical evidences indicate the potential of conductivity imaging to add a valuable dimension to MRI‐based tissue assessment. By quantifying the electrical properties of living tissue, conductivity imaging bridges the structural, functional, and biophysical imaging domains, advancing precision diagnosis and personalized therapy.
